# The Diagnosis and Treatment of Pneumonia in Very Young Infants

**Published:** 1891-04

**Authors:** 


### Selections and Abstracts

THE DIAGNOSIS AND TREATMENT OF PNEUMONIA IN VERY YOUNG INFANTS, is the title of a paper read before the Philadelphia Obstetrical Society, Nov. 6, 1890, by Dr. B. C. Hirst.

He considers pneumonia the most frequent of all the serious diseases in the first week or so of infantile life. He questions whether exposure to cold, except in premature infants, can be considered as an etiological factor, but regards inspiration of foreign bodies, as blood meconium or vaginal discharges, during or immediately after labor, as the almost invariable cause.

The most important diagnostic features of these are:

1st. High temperature, when this is met with in the first week or so, is very significant—although constant in pneumonia, we should not forget the possibility of septic infection of the umbilical cord, which may take place when to all external appearances the cord, or stump, may seem healthy.

2d. Rapid breathing is a very striking symptom. He relates a case with recovery, in which the respiration reached 127 per minute—also states that in some feeble premature infants, that there may be pneumonia without acceleration of respiration, and very little rise of temperature.

3d. Cough. This is a variable symptom, and may or may not be present.

4th. History of Dystocia. This he considers of some value, stating that a child will be more likely to make efforts at inspiration in a difficult than in an easy labor during its passage through the parturient tract.

5th. Physical signs in the chest. These he thinks are very difficult if not impossible to clearly define in many cases.

Treatment. General, cardiac and respiratory and the relief of internal congestion.

1st. Proper food at regular intervals. 2d. Medication, which consists of carb. ammon, digital. and brandy. 3d. Poultices and counter irritation.

During the discussion, the following points were brought out: Dilatation and contraction of the alea of the nose with each respiration, also the tendency for the child to raise its hand to the ear. Both of these were spoken of as symptoms of some importance—also the favorable action of syr. scilla co. in these cases.—*Annals of Gynecology and Pediatry, Dce., 1890.*

